# Use of tourniquet in a sickle cell patient for sequestrectomy and saucerisation: a case report

**DOI:** 10.1186/1757-1626-2-9085

**Published:** 2009-11-25

**Authors:** Abiodun Oyinpreye Jasper

**Affiliations:** 1 Department of Anaesthesia, College of Health Sciences, Delta state University, Abraka Delta State, Nigeria

## Abstract

A 25 yr old 60 kg haemoglobin SS male presented with a 3 year history of a discharging sinus in the left upper arm. His last crisis was ten years earlier; while his stable haemoglobin concentration was 10 g/dl.

Examination revealed healthy looking, well hydrated man. He had left upper arm chronic osteomyelitis which was diagnosed radiologically. He was scheduled for an elective saucerization and sequestrectomy. Tourniquet was applied to reduce blood loss and maintain a relatively bloodless field at surgery; and nitrous oxide/oxygen/halothane relaxant technique of anaesthesia was adopted.

No perioperative problems were encountered and the patient made remarkable improvement and was discharged home. At post surgical review a few weeks after, he was stable and well.

## Introduction

Sickle cell disease is a family of haemoglobin disorders with autosomal dominance inheritance [1] the beta chain of haemoglobin has valine substituted for glutamine in position 6 of the normal adult haemoglobin.

Generally, sickle cell disease is found in Negroes and non-Negroes race in the African continent, Mediterranean and in parts of India. The prevalence in Nigeria is about 0.4-3%[2]. In the homozygous form, red blood cells lose their normal morphology and become sickle-shaped if they are exposed to hypoxia, acidosis, low temperature or cellular dehydration [3]

Anaesthesia for homozygous sickle cell patients can be very challenging. The anaesthetist is confronted with the problems of anaemia, which may necessitate reduction of intra-operative blood loss by the application of tourniquet (which on its own pre-disposes the patient to a sickle cell crisis).

## Case report

E.I. a 25 yr old sickler, weighing 60 kg, presented in the orthopaedic clinic with a 3-year history of discharging sinus in his left upper arm. He was diagnosed as a sickle cell (Hb SS) patient many years earlier though his physical features were not suggestive of haemoglobin SS disease. The last episode of a crisis was 10 yrs before presentation.

His pulse rate was 100 beats/minute, regular with normal volume. While the blood pressure was 120/80 mmHg [26/10.7 Kpa]. The heart sounds were normal. His packed cell volume was 30%. Kidney and liver function tests were essentially normal. The X-ray of the left arm showed circumscribed areas of increased radiodensity; with areas of increased radioluscency in the middle third of left humerus-suggestive of osteomyelitis. He has had Ampiclox capsules and Erythromycin tablets at different times since the onset of symptoms. His airway was assessed, as Mallam pati I while the anaesthetic risk assessment was ASA II. His premedication drug was oral diazepam (10 mg) at night and 10 mg on morning of surgery, and he was generally calm at induction. Monitors attached to the patient were pulse oximeter, thermocouple and a sphygmomanometer. The urine output was monitored with urethral catheter and bag.

500 mls of warm normal saline was infused before induction of anaesthesia. The patient was pre-oxygenated for 5 minutes. 15 mg of pentazocine was given along with 8 mgs of pancuronium (as a mega dose) to shorten time of endotracheal intubation. Suxamethonium was not used. 300 mg of thiopentone was given in divided doses of 100 mg over 90 sec from the point of administration of pancuronium, for induction of anaesthesia. He was ventilated by a facemask with 100% oxygen using Malpeson A breathing system. On full muscle relaxation, the trachea was intubated with a size 8.00 mmID polyvinyl endotracheal tube and connected to a closed circuit with soda lime absorber. The lungs were manually ventilated using intermittent positive pressure ventilation with 50% nitrous oxide and oxygen in 0.5% of halothane

Further, his body was well covered to minimize heat loss. Vital signs remained relatively stable with monitoring of temperature, pulse rate, blood pressure and oxygen saturation. The tourniquet time was 1 hour. Blood loss was 500-mls and 2 litres of warms crystalloids were given to the patient for replacement and fluid maintenance. Oxygen saturation was 99-100% throughout the surgery and urine output was adequate.

At the end of surgery, residual muscle relaxation was reversed safely. The patient was transferred to the recovery room. The patient had supplemental oxygen for 24 hours at a rate of 5 liters min^-1 ^by facemask. Fluid intake was 1 litre of 4.3% dextrose saline, alternating with 1 litre of 5% dextrose 8 hourly over 24 hours. Analgesia was provided with pentazocine 30 mg i.m 8 hourly for 48 hours. This was followed by diclofenac sodium and 500 mg of oral Cefuroxime for 10 days.

He was discharged home, by the 6^th ^postoperative day with packed cell volume (PCV) of 28%; on haematinics.

## Discussion

In sickle cell (homozygote SS), the abnormal gene is due to substitution of valine for glutamic acid at position 6 of the beta-chain of haemoglobin A. 90% of haemoglobin in the red blood cell of the homozygote SS is S[1]. Haemoglobin S is vulnerable to low oxygen tension. At 5.3 Kpa (40 mmHg), the reduced haemoglobin forms complex strands called 'tactoids" which distort the red cells to form the characteristic sickle shape. The molecular basis of this is aggregation of oxygenated haemoglobin molecules along their longitudinal axis [4]. Sickle cells are fragile, and haemolyse; they also block small vessels resulting in infarction. The resultant cell death and tissue infarction at the site of obstruction is termed a sickle cell crisis.

Although some sicklers are asymptomatic, possibly due to their ability to maintain a higher than normal haemoglobin F throughout their lives, majority are not so fortunate. Many episodes of sickling may occur spontaneously, although certain factors may increase the risk [2]. Such factors include hypoxia, hypothermia, tourniquets, dehydration, acidosis, circulatory stasis and pyrexia. Patients with sickle cell disease usually have an associated history of poor development and failure to thrive. Sickling and microvascular occlusion within bones and epiphyseal plates often predispose such patients to limb shortening, joint deformity and osteomyelitis (as was occasioned in the patient) Chronic osteomyelitis was diagnosed based on the clinical features and was confirmed radiologically.

In Nigeria, sickle cell patients may be accepted for surgery with a haemogbolin of 6-10 gm.dL^-1 ^[5] however, in the Western world the story is not the same. Although it is accepted that preoperative transfusion can be avoided in sicklers having low risk surgery, the recommendation is that transfusion should be done for high risk surgery [6]. Low risk surgery, applies to procedures not associated with significant blood loss while high risk surgery implies procedures associated with a potential for significant blood loss. Procedures on the bone tend to bleed intraoperatively and ooze postoperatively because homeostasis may be difficult, thus suggesting that sequestrectomy is a potential high risk surgery.

Hypovolaemia must be avoided to prevent dehydration, hyperosmolality and increased viscosity, by giving intravenous fluid therapy during the period of preoperative starvation. Goodwin [7] suggested preoperative hydration for at least 8 hours of intravenous fluids for high risk cases and either intravenous hydration or oral hydration with clear fluids until 3-4 hours preoperatively for low risk cases [7]. The patient received a maintenance dose of 4 ml.kg-1 hr-1 of 5% dextrose in saline over 6 hours (about 1 litre of fluids

Desired monitoring should include pulse oximetry, temperature, urine output and the state of hydration with a central venous line. Blood pressure measurement, electrocardiogram and tidal carbon dioxide are also desirable. In the absence of most of these equipments, those available appeared to suffice. Adequate draping and use of warmed fluids were applied to reduce hypothermia.

In the event of hypotension developing introperatively, plasma volume expanders may be used for treatment. In sicklers, crystalloids should be used with caution because of the risk of fluid overload avoided except in resistant cases of hypotension where small doses should be used to avoid the exacerbation of a crisis [8]. Supplemented oxygen (100%) by facemask was given intraoperatively to improve the patient's oxygen content to reduce the incidence of sickling. This extended into the postoperative period

A tourniquet was used intraoperatively to further guarantee minimal blood loss. Controversies govern the use of tourniquets. It has been reported that the use of tourniquets may produce local stasis and acidosis which may enhance sickling [9]. Also, Oginni in his study noted a significantly higher incidence of complications in sickle cell patients than in a normal group[10]; while other workers have concluded that prolonged use of tourniquets in patients with sickle cell disease is safe, provided that optimum acid - base status and oxygenation are maintained throughout the procedure [11,12];. Facilities for blood analysis were not available in this patient. Where acidosis occurs, a period of hyperventilation or administration of sodium bicarbonate prior to release of tourniquet may be sufficient[1].

Although autologous blood transfusion is now possible with the use of intraoperative cell saver for patients with sickle cell disease [12], this patient was not considered for such a procedure because the blood lost was infected with sequestrum. Blood was not transfused during surgery as the patient's blood loss 500 ml, was thought to be modest. Oxygen therapy was not continued beyond the first 24 hours postoperatively. While it was beneficial in preventing postoperative hypoxia, its continuous use may cause depression of erythropoiesis, so that anaemia is worsened later [1].

Parenteral pentazocine 0.6 mg.kg^-1 ^(30 mg) in 8 hourly over 48 hours appeared to suffice without any associated cardio respiratory depression. This was followed up with diclofenac orally and topical piroxicam to achieve adequate analgesia. In the post operative period, adequate hydration and analgesia was maintained to prevent vaso-occlusive crisis. Postoperative vaso-occlusive crisis have been associated with 12% mortality [13]. Postoperative antibiotics were also given to treat infection and reduce the likelihood of sickling.

## Conclusion

Sickle cell disease is associated with chronic anaemia and a predilection to crisis development. Avoidance of stress producing factors like dehydration, hypoxia, hypothermia, acidosis, circulatory stasis and pyrexia are helpful in reducing crisis rate. The use of tourniquet is still controversial. Careful preoperative preparation and anaesthesia lead to a successful outcome in most circumstances.

## Abbreviations

Hb SS: Haemoglobin SS; ASA: American Society of Anesthesiology; IV: intravenous; PCV: Packed Cell Volume.

## Consent

Written informed consent was obtained from the patient for the publication of this case report.

## Competing interests

The author declares that they have no competing interests.
